# Prognostic Significance of High VEGF-C Expression for Patients with Breast Cancer: An Update Meta Analysis

**DOI:** 10.1371/journal.pone.0165725

**Published:** 2016-11-03

**Authors:** Zhiqiao Zhang, Guanying Luo, Hongfeng Tang, Canchang Cheng, Peng Wang

**Affiliations:** 1 Department of Infectious Disease, The First People’s Hospital of Shunde, Shunde, Guangdong, China; 2 Department of Internal Medicine, The Chencun Affiliated Hospital of First People’s Hospital of Shunde, Shunde, Guangdong, China; 3 Department of Science and Education, The First People’s Hospital of Shunde, Shunde, Guangdong, China; University of North Carolina at Chapel Hill School of Medicine, UNITED STATES

## Abstract

**Background:**

The prognostic significance of vascular endothelial growth factor C (VEGF-C) expression in breast cancer (BC) patients remains controversial. Therefore, this meta-analysis was performed to determine the prognostic significance of VEGF-C expression in BC patients.

**Materials and Methods:**

Several electronic databases were searched from January 1991 to August 2016. The pooled hazard ratios (HRs) and 95% confidence intervals (CIs) were calculated to evaluate the prognostic significance of VEGF-C expression for disease free survival (DFS) and overall survival (OS).

**Results:**

The present meta analysis totally included 21 eligible studies and 2828 patients with BC. The combined HRs were 1.87(95% CI 1.25–2.79, *P* = 0.001) for DFS and 1.96(95% CI 1.15–3.31, *P* = 0.001) for OS. The pooled HRs of non-Asian subgroup were 2.04(95%CI 1.36–3.05, *P* = 0.001) for DFS and 2.61(95%CI 1.51–4.52, *P* = 0.001) for OS, which were significantly higher than that of Asian subgroup. The funnel plot for publication bias was symmetrical. The further Egger's test and Begg's test did not detect significant publication bias (all *P*>0.05).

**Conclusions:**

The present meta analysis strongly supported the prognostic role of VEGF-C expression for DFS and OS in BC patients, especially for patients in non-Asian countries. Furthermore, stratification by VEGF-C expression may help to optimize the treatments and the integrated managements for BC patients.

## Introduction

Breast cancer (BC) is the most common malignant tumor and the leading cause of cancer death in females worldwide, resulting in 14% of the total cancer deaths in 2008 [1]. Breast cancer is a complicated tumor with different clinical characteristics and poor prognosis. Several clinical and pathological features, including HER-2 status, histological grade, and hormone receptor status, have been used to predict the treatment response and clinical prognosis in BC patients[2–3]. However, these factors are insufficient to accurately predict poor prognosis for BC patients. Therefore, it is necessary to find a reliable prognostic factor to predict the clinical prognosis for BC patients.

As a member of VEGF family, vascular endothelial growth factor C (VEGF-C) is an important influence factor for angiogenesis and lymphangiogenesis in tumors. It has been reported that VEGF-C is commonly expressed in breast cancer[4].So far, the relationship between VEGF-C expression and prognosis of BC patients was still contradictory in various original studies [5–25]. Several studies reported that high VEGF-C expression had a significant correlation with poor survival in BC patients [17, 19, 24]. On the contrary, some studies reported that high VEGF-C expression had a significant association with favorable survival for BC patients [13, 22]. More interestingly, four relevant meta analyses provided two opposite opinions on relationship between high VEGF-C expression and clinical prognosis in BC patients [26–29]. The contradiction among these original studies and meta analyses seriously hindered the clinical utility of VEGF-C expression in BC patients. Therefore, we performed this meta-analysis to further clarify the prognostic significance and clinical value of VEGF-C expression for BC patients.

## Materials and Methods

### Literature search strategy

Several electronic databases, including EMBASE, PubMed, Web of Knowledge, and Cochrane Library, were searched from January 1991 to August 2016. We performed literature search by combining MeSH term and text word in PubMed databases with the follow terms: "VEGF-C" or "vascular endothelial growth factor C " and "breast" or " mammary " and "cancer" or "carcinoma" or "tumor" and "survival" or "outcome" or "prognosis" or "prognostic". The search strategies for EMBASE and other databases were similar but were adapted correspondingly. Expanded search of hyponym was performed in the retrieval process. In addition, we performed a manual search according to the references of the relevant articles to supplement eligible studies. If necessary, we even contacted the corresponding author to get necessary information. The search was restricted to human studies, but there were no restrictions on language or publication time. All clinical investigation and data achievement were performed according to the principles of Declaration of Helsinki.

### Criteria for inclusion and exclusion

The inclusion criteria of eligible studies were as follows: (1) proven pathological diagnosis of BC in humans; (2) enough information of overall survival such as hazard ratio (HR) and 95% confidence interval (CI). Studies not directly providing hazard ratio and 95% confidence interval were included if survival information were available from curves or tables for statistical estimation of HR. Articles published in Chinese were included in this meta-analysis as English literature. For multiple studies from the same population, only the most recently published study was included in this meta-analysis.

The following studies were excluded: (1) laboratory studies; (2) non-human experiments; (3) reviews, letters, case reports, and conference abstracts without original data; (4) lack of the necessary survival data.

### Quality assessment of studies

Two reviewers (Zhiqiao Zhang and Hongfeng Tang) independently evaluated the quality of the studies included in the present meta analysis by using Newcastle-Ottawa Quality Assessment Scale (NOS). The NOS comprises assessments of patient selection, study comparability, follow-up, and outcome of interest. The total scores were used to assess the study quality. Disagreements between two reviewers were resolved through consensus with another reviewer (Guanying Luo).

### Data extraction

Two investigators (Zhiqiao Zhang and Hongfeng Tang) independently extracted the following data from the original studies: surname of the first author, detection method of VEGF-C expression, patient number, region, publication year, disease stage, clinical parameters, and survival outcome data (HRs and 95%CIs). The information were extracted and recorded by using a standardized form. All eligible studies were coded as surname of the first author + publish year in the standardized form. Study authors were contacted to get key information if necessary. Disagreements between two investigators were resolved by discussion. If necessary, a third investigator (Guanying Luo) helped to reach a consensus.

### Statistical analysis

The statistical analysis was performed according to the suggestions of the Meta-Analysis of Observational Studies in Epidemiology group (MOOSE)[30]. The hazard ratios (HRs) and 95% CIs were used to summary outcome of overall survival. We directly obtained pooled HRs and 95% CIs if the statistical data were reported in the study. While HRs and 95% CIs were not directly reported in the studies, survival information was extracted from Kaplan-Meier curve and used to estimate HR. The heterogeneity was evaluated using *I*^2^ statistic, which was defined according to the Cochrane Handbook [31]: 0% to 40%, negligible heterogeneity; 30% to 60%, moderate heterogeneity; 50% to 90%, substantial heterogeneity; 75% to 100%, considerable heterogeneity. The subsequently meta-analysis was performed using random effect model with DerSimonian and Laird method[32], which applying the inverse of variance as a weighing factor. Meta-regression analyses with REstricted Maximum Likelihood (REML) method and subgroup analyses were performed to explore the sources of heterogeneity. Funnel plot, Begg's test[33], and Egger's test[34] were employed to evaluate publication bias. *P* value<0.05 was considered statistically significant. The statistical analyses were performed by STATA version 12.0 software (Stata Corporation, College Station, Texas, USA).

## Results

### Search Results

**Fig 1:**
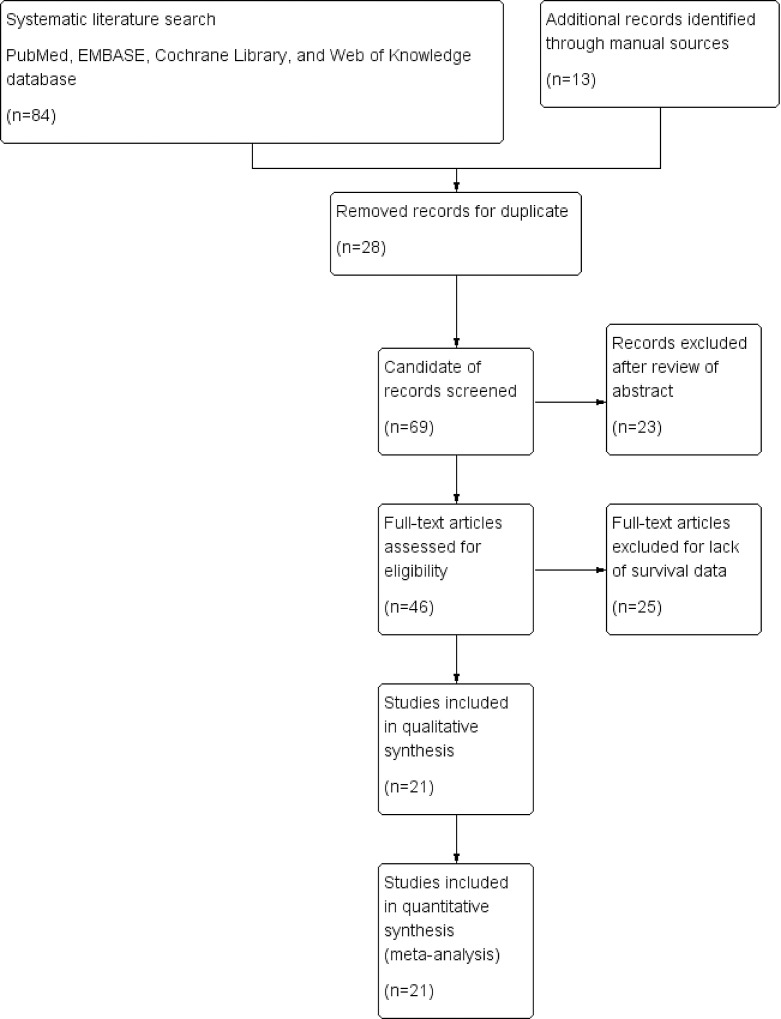
Flowchart of study selection in present meta-analysis.

The initial search found a total of 97 articles (with 28 duplicate articles). After reviewing the abstracts, 23 irrelevant articles were excluded according to the criteria for inclusion and exclusion. Reviewers identified 46 potential studies for full-text review and 25 articles were eliminated due to inadequate survival data. Finally, 21 eligible studies were included in the present meta-analysis [5–25]. The details of search process were summarized in Fig 1. Quality assessment of 21 involved studies were assessed by using the Newcastle-Ottawa Scale (NOS).

### Study selection and characteristics

The characteristic of the 21 included studies were summarized in Table 1. The publication time of the included studies ranged from 2001 to 2015.The patient number of 21 studies ranged from 61 to 377, with a mean sample size of 135. The mean time of follow-up period ranged from 32 to 135 months. The NOS scores of 21 included studies varied from 7 to 8, with a mean value of 7.1. VEGF-C expression was measured in surgical tumor tissues. All studies provided enough information of survival data and/or survival curve.

**Table 1:** Characteristics of studies included in present meta analysis.

			Cutoff	Total	Positive	Age	History			Survival	Data	NOS
Study	Country	Method	Level	Number	(%)	(range)	Type	Stage	Therapy	Outcome	Extraction	Value
Zhang 2006	China	PCR	Median	61	67.4	52.3(28–75)	BC	NR	S	DFS	Curve	7
Linardou 2012	Greece	PCR	75.0%	167	24.9	50(22–78)	BC	NR	S+C	DFS/OS	Curve	8
Watanabe 2005	Japan	IHC	Score≥2+	87	43.7	53.5±14.4	BC	NR	S+C	DFS/OS	Curve	8
Yang 2001	China	IHC	Median	107	50	52(33–77)	BC	NR	S+C	DFS	Curve	7
Zhao 2012	China	IHC	Score≥4	78	47.4	54(29–75)	BC	I-III	S+C	DFS/OS	Curve	7
Cao 2003	China	IHC	Score≥3	66	66.6	49(29–77)	BC	I-III	S	OS	Curve	7
Li 2009	China	IHC	Score≥2+	117	58.1	51(29–74)	BC	I-III	S+C	DFS	Curve	7
Liu 2013	China	IHC	Score≥1+	116	50.9	52(32–77)	BC	I-III	S	DFS/OS	Curve	7
Bando 2006	Japan	ELISA	Score≥0.326	193	54.4	54(30–86)	BC	NR	S+C	DFS/OS	Reported	7
Gisterel 2010	Poland	PCR	≥1784	377	NR	57(29–83)	BC	I-III	S+C	DFS	Reported	7
Mohammed 2007	England	IHC	Median	177	37	57(32–70)	BC	I-II	S	DFS/OS	Reported	7
Tsutsui 2010	Japan	IHC	Score≥5	242	78	58(23–86)	BC	I-III	S+C	DFS	Reported	7
Gu 2008	China	IHC	Score≥2+	61	70.5	58(29–90)	BC	I-III	S+C	DFS/OS	Reported	7
Kinoshita 2001	Japan	IHC	≥10%	98	39.8	55(30–86)	BC	I-IV	S+C	DFS	Reported	7
Mylona 2007	Greece	IHC	≥10%	177	48	57(25–86)	BC	I-III	S	DFS/OS	Reported	7
Nakamura 2006	Japan	IHC	Score≥5	113	82.3	NR	BC	I-III	S+C	OS	Reported	7
Nakamura 2003	Japan	IHC	≥10%	103	83.7	51(24–87)	BC	I-III	S	DFS	Reported	7
Ni 2013	China	IHC	NR	75	64	NR	BC	I-III	S+C	OS	Reported	7
Yang 2015	China	IHC	≥10%	218	62.3	NR	BC	I-III	S	OS	Reported	7
Zhang 2008	China	IHC	Score≥2+	70	42.9	49(30–77)	BC	I-III	S	DFS/OS	Reported	7
Zhou 2004	China	IHC	≥25%	125	47.8	48(23–78)	BC	I-III	S	OS	Reported	7

Note: NR, not reported; OS, overall survival; DFS, disease free survival; S, surgery; C, chemotherapy; NOS, Newcastle-Ottawa Quality Assessment Scale. Age was presented as median (range); IHC, immunohistochemistry; ELISA, enzyme linked immunosorbent assay; PCR, polymerase chain reaction; BC, breast cancer.

### Prognostic significance of high VEGF-C expression in BC patients

**Fig 2:**
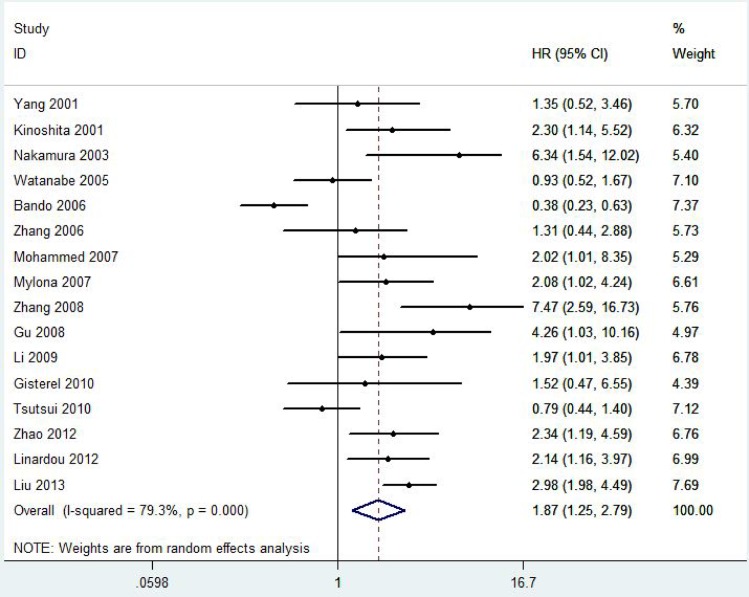
Forest plot diagrams of hazard ratios for correlations between VEGF-C expression and DFS.

**Fig 3:**
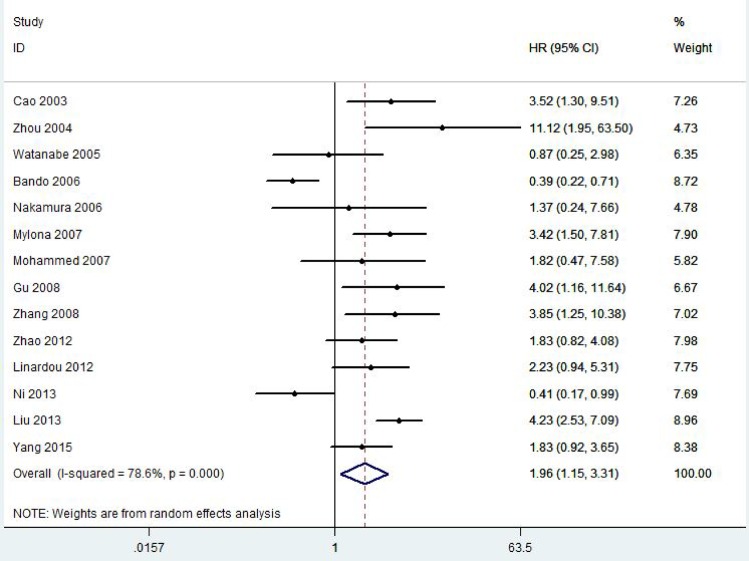
Forest plot diagrams of hazard ratios for correlations between VEGF-C expression and OS.

A total of 2828 patients with BC from 21 eligible studies were included and analyzed for prognostic significance of VEGF-C expression (Fig 2 and Fig 3). The combined HRs were 1.87(95% CI 1.25–2.79, *P* = 0.001) for DFS and 1.96(95% CI 1.15–3.31, *P* = 0.001) for OS in BC patients.

### Publication bias

**Fig 4:**
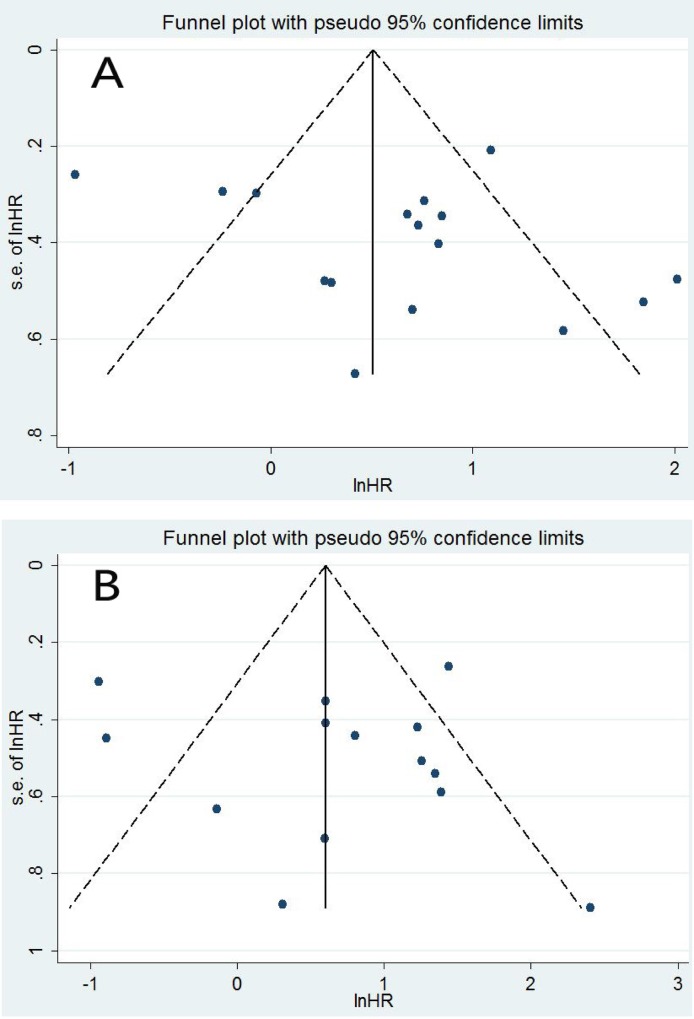
**Funnel plot for all eligible studies which provided HRs of high VEGF-C expression for DFS (A) and OS (B).**

The funnel plot, Begg's test, and Egger's test were further performed to assess the publication bias. The funnel plot for publication bias was symmetrical for both DFS and OS (Fig 4). There was not significant publication bias according to Egger's test (*P* = 0.275) and Begg's test (*P* = 0.392) in the present study for DFS. Similarly, the publication bias was not significant for OS according to Egger's test (*P* = 0.646) and Begg's test (*P* = 0.913).

### Subgroup analyses and meta-regression analyses

Subgroup analyses and meta-regression analyses were further performed to explore the sources of heterogeneity. Subgroup analyses (Table 2) demonstrated that regions and treatments might contribute to the clinical heterogeneity. Further meta-regression analysis suggested that treatments might be a potential source of heterogeneity for DFS (*P* = 0.045) and for OS (*P* = 0.017).

**Table 2:** Subgroup analyses for association between VEGF-C expression and survival in BC patients.

Group factors	Subgroup	Study	HR	LCI	HCI	*P* value	*I*^2^	*P* value
DFS								
Patients number≥100	Yes	10	1.65	0.97	2.83	0.067	83.3	0.001
	No	6	2.3	1.25	4.24	0.007	70.5	0.005
Regions	Asian	12	1.86	1.12	3.1	0.017	84.6	0.001
	Non- Asian	4	2.04	1.36	3.05	0.001	0	0.975
IHC method	Yes	12	2.2	1.51	3.21	0.001	68.7	0.001
	No	4	1.09	0.41	2.9	0.869	84.8	0.001
Treatments	S	6	2.97	1.87	4.71	0.001	51.1	0.069
	S+C	10	1.41	0.88	2.27	0.151	76.9	0.001
Positive rate≥50%	Yes	9	1.64	0.87	3.1	0.126	85.4	0.001
	No	7	2.18	1.41	3.37	0.001	58.8	0.024
OS								
Patients number≥100	Yes	8	2.11	0.99	4.5	0.053	83.7	0.001
	No	6	1.78	0.83	3.85	0.140	71.7	0.003
Regions	Asian	11	1.85	0.96	3.55	0.064	82.7	0.001
	Non- Asian	3	2.61	1.51	4.52	0.001	0	0.670
IHC method	Yes	12	2.28	1.43	3.62	0.001	63.4	0.002
	No	2	0.91	0.16	4.99	0.910	90.6	0.001
Treatments	S	7	3.3	2.37	4.6	0.001	6	0.382
	S+C	7	1.12	0.54	2.32	0.756	75.6	0.001
Positive rate≥50%	Yes	7	1.55	0.64	3.75	0.327	87.9	0.001
	No	7	2.46	1.58	3.83	0.001	21.4	0.266

HR, hazard ratio; CI, confidence interval; LCI, lower value of confidence interval; HCI, higher value of confidence interval; S, surgery; C, chemotherapy; OS, overall survival; DFS, disease free survival.

For DFS, the pooled HR of non-Asian subgroup was 2.04(95%CI 1.36–3.05, *P* = 0.001), which was higher than that of Asian subgroup. Similarly, the pooled HR for non-Asian subgroup for OS was 2.61(95%CI 1.51–4.52, *P* = 0.001), which was significantly higher than 1.85(95%CI 0.96–3.55, *P* = 0.064) for Asian subgroup, suggesting that high VEGF-C expression might be more closely associated with poor survival for BC patients in non-Asian countries.

The pooled HRs of IHC subgroup were 2.20(95%CI 1.51–3.21, *P*< 0.001) and 2.28(95%CI 1.43–3.62, *P*< 0.001) for DFS and OS respectively, which were significantly higher than that of non-IHC subgroup.

### Sensitivity analyses

All studies were sequentially removed to assess that whether or not any individual study had a significant influence to the pooled HRs. The pooled HRs of sensitivity analyses varied from 1.71(95%CI: 1.15–2.53) to 2.08 (95%CI: 1.52–2.85) for DFS, demonstrating that the pooled HRs were not significantly influenced by any individual study for DFS(Table 3). Similarly, the pooled HRs for OS ranged from 1.86(95%CI: 1.07–3.23) to 2.27 (95%CI: 1.45–2.51), suggesting that the results of the present meta analysis was stable and reliable.

**Table 3:** Effect of individual studies on the pooled HRs of VEGF-C expression for DFS and OS.

	DFS			OS		
Study omitted	HR	LCI	HCI	HR	LCI	HCI
1	1.9083867	1.2520173	2.90886	1.9550176	1.1543682	3.3109832
2	1.8456045	1.2074366	2.82106	1.9550176	1.1543682	3.3109832
3	1.7387738	1.164133	2.59707	1.8697225	1.0708288	3.2646322
4	1.9721094	1.2912425	3.01199	1.9550176	1.1543682	3.3109832
5	2.0827684	1.5197888	2.85429	1.7930649	1.0554458	3.0461838
6	1.9120828	1.2544384	2.9145	2.0678508	1.1913106	3.58933
7	1.8624746	1.2243328	2.83323	1.9550176	1.1543682	3.3109832
8	1.8585508	1.2116612	2.85081	2.272918	1.489395	3.4686273
9	1.7095664	1.1548227	2.53079	1.9931914	1.151803	3.4492115
10	1.7887729	1.1859893	2.69792	1.8671988	1.0640525	3.2765595
11	1.8663094	1.213935	2.86927	1.9668693	1.1298384	3.4240075
12	1.8880523	1.2454731	2.86216	1.8583227	1.0703709	3.226324
13	1.9948787	1.317013	3.02164	1.859373	1.0684728	3.2357099
14	1.8423521	1.2013937	2.82527	1.9550176	1.1543682	3.3109832
15	1.8550151	1.2047843	2.85618			
16	1.7989184	1.1788964	2.74503			
Combined	1.8677404	1.2509044	2.78875	1.9550176	1.1543682	3.3109832

HR, hazard ratio; CI, confidence interval; LCI, lower value of 95% CI; HCI, high value of 95% CI; OS, overall survival; DFS, disease free survival.

### Stability assessment of the pooled HRs of VEGF-C expression for survival by cumulative meta-analyses

**Fig 5:**
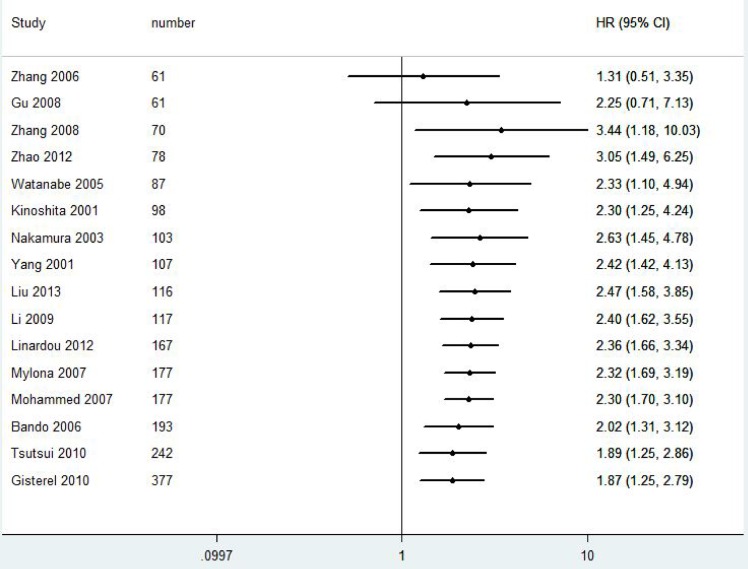
Cumulative meta-analyses for stability of the pooled HRs of VEGF-C expression for DFS in BC patients.

**Fig 6:**
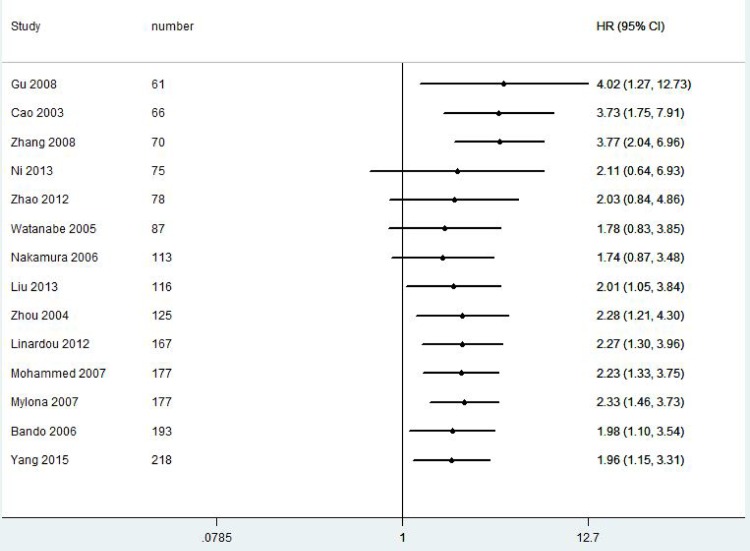
Cumulative meta-analyses for stability of the pooled HRs of VEGF-C expression for OS in BC patients.

We further performed cumulative meta-analyses to determine the stability of VEGF-C expression for survival in BC patients (Fig 5 and Fig 6).With inclusions of studies that patient number more than 70, the pooled HRs for DFS ranged from 1.89 to 3.44. The pooled HRs for OS varied from 1.98 to 2.33 with inclusions of studies that patient number more than 116, indicating that the prognostic significance of VEGF-C expression for survival in BC patients was stable.

## Discussion

The present meta analysis demonstrated that high VEGF-C expression is significantly associated with poor survival in BC patients. The combined HRs were 1.87 for DFS and 1.96 for OS in BC patients. The pooled HRs of non-Asian subgroup were 2.04 for DFS and 2.61 for OS, which were significantly higher that of Asian subgroup. Further sensitivity analyses demonstrated that the relation between high VEGF-C expression and poor prognosis of BC patients did not change after removing any individual study. Furthermore, cumulative meta-analyses also demonstrated that the predictive value of VEGF-C expression for prognosis of BC patients was stable and reliable.

There were two opposite opinions in four previous meta analyses [26–29]. In 2012, Wang J et al. reported that VEGF-C expression could predict poor prognosis in BC patients, with a pooled HR of 2.164 (95% CI 1.256–3.729) for DFS and a pooled HR of 2.613 (95% CI 1.256–3.729) for OS [26]. In 2014, Liang B et al. reported that VEGF-C expression was significantly associated with poor OS (OR = 2.46, 95% CI 1.46–4.14) and DFS (OR = 2.10, 95% CI 1.32–3.35) in BC patients [27]. By contrast, Gao S et al. reported that high VEGF-C expression was not associated with poor DFS (HR = 0.80, 95% CI 0.51–1.51) or OS (HR = 1.08, 95% CI 0.37–1.78) in BC patients in 2014[28]. In 2016, Wang F et al. also reported that there was no significant association between high VEGF-C expression and OS (HR = 0.76, 95% CI 0.43–1.33) in BC patients [29]. The contradictory conclusions in different meta analyses leaded to great confusion on whether or not high VEGF-C expression was associated with prognosis in BC patients. Remarkably, according to Cochrane handbook for meta analysis, odds ratio is not suitable for survival analysis with time-to-event data in consideration of censored data and time to study endpoint. Meanwhile, one original study [35] included in the meta analysis performed by Wang J et al. did not extract the right survival data.

The conclusions of the current meta analysis was generally similar to that of two previous meta analyses performed by Wang J et al. and Liang B et al.[26–27]. Compared with four previous meta analyses above, the present meta analysis had six strengths which provided powerful support to the conclusions in the present meta analysis. Firstly, the present meta analysis totally included 21 eligible studies and 2828 BC patients. The numbers of studies and patients were significantly more than that of the previous meta analyses and could significantly increase persuasiveness of the conclusions. Secondly, sensitivity analyses demonstrated that the pooled HRs were not significantly affected by any individual study. Thirdly, cumulative meta-analyses provided reliable evidences to support the final conclusions. Fourthly, subgroup analysis further demonstrated that the pooled HRs of non-Asian subgroup were significantly higher than that of Asian subgroup for both DFS and OS, suggesting that high VEGF-C expression might be more closely associated with poor survival of BC patients in non-Asian countries. Fifthly, considering that detection method might be a source of clinical heterogeneity and affect the pooled HR, we conducted a comparison between IHC subgroup and non-IHC subgroup. The comparison results demonstrated that the pooled HRs of IHC subgroup were significantly higher than that of non-IHC subgroup for both DFS and OS, suggesting that prognostic significance of VEGF-C expression for prognosis of BC patients was stable and reliable by using IHC method. Sixthly, studies published in Chinese were also included in the present meta analysis as English studies, increasing the representation of the included studies. These six strengths significantly enhanced persuasive power of the conclusions in the present study. In addition, some meta analyses have explored the clinical value of VEGF-C expression as a predictive tool for prognosis in different tumors, such as colorectal cancer and non-small cell lung cancer [36–37].

The heterogeneity was significant in the present meta analysis. The heterogeneity in the present meta analysis might be caused by the following reasons. First, both subgroup analyses and meta regression analyses showed that treatments might be a potential source of clinical heterogeneity. Second, there was no significant heterogeneity in non-Asian subgroup by subgroup analyses, indicating that regions might be a potential source of heterogeneity. Third, the true heterogeneity might be caused by differences in the intensity of interventions or differences in underlying risk between studies with different sizes.

Publication bias is important for interpreting the conclusions. The funnel plot for publication bias was symmetrical in the present meta analysis. The further Egger's test and Begg's test did not detect significant publication bias (all *P*>0.05), suggesting that the results might not be influenced by the publication bias.

The conclusions of the present meta analysis should be interpreted cautiously for the following reasons: First, the positive status of VEGF-C expression was defined according to different cut-off values in various studies, which might result in clinical heterogeneity. Second, different baseline characteristics, such as tumor stages and races, might result in clinical heterogeneity and reduce the persuasiveness of the conclusions in the current meta analysis.

In conclusion, the present meta analysis strongly supported the prognostic role of VEGF-C expression for DFS and OS in BC patients. Furthermore, stratification by VEGF-C expression may help to optimize the treatments and the integrated managements for BC patients.

## Supporting Information

S1 File This is the S1 File which is the original statistical document for the meta analysis(Part1). (DTA)

S2 File This is the S2 File which is the original statistical document for the meta analysis(Part2). (DTA)

S3 File This is the PRISMA checklist. (DOC)

S4 File This is the PLOSOne_Clinical_Studies_Checklist. (DOCX)
